# Engineering photosynthetic organisms for the production of biohydrogen

**DOI:** 10.1007/s11120-014-9991-x

**Published:** 2014-03-27

**Authors:** Alexandra Dubini, Maria L. Ghirardi

**Affiliations:** National Renewable Energy Laboratory, 15013 Denver West Parkway, Mail Box 3313, Golden, CO 80401 USA

**Keywords:** Green algae, H_2_ metabolism, Hydrogenases, Electron transfer, Genetic engineering

## Abstract

Oxygenic photosynthetic organisms such as green algae are capable of absorbing sunlight and converting the chemical energy into hydrogen gas. This process takes advantage of the photosynthetic apparatus of these organisms which links water oxidation to H_2_ production. Biological H_2_ has therefore the potential to be an alternative fuel of the future and shows great promise for generating large scale sustainable energy. Microalgae are able to produce H_2_ under light anoxic or dark anoxic condition by activating 3 different pathways that utilize the hydrogenases as catalysts. In this review, we highlight the principal barriers that prevent hydrogen production in green algae and how those limitations are being addressed, through metabolic and genetic engineering.  We also discuss the major challenges and bottlenecks facing the development of future commercial algal photobiological systems for H_2_ production. Finally we provide suggestions for future strategies and potential new techniques to be developed towards an integrated system with optimized hydrogen production.

## H_2_ energy carrier

Microalgae have gained relevance recently as versatile organisms that are able to harvest solar energy and convert it into a variety of products of commercial significance, from nutraceuticals to fuels. One of the useful products of algal metabolism is the energy carrier hydrogen (H_2_). Besides being the third most abundant element on the earth, H_2_ can be produced by a variety of sustainable technologies and can be easily interconverted into electricity for storage and transport. One of the major advantages of H_2_ as an energy carrier is the fact that its combustion does not release toxic products. Available technologies for production of H_2_ gas mostly involve reforming methanol. However, sustainable methods to extract H_2_ from water through photocatalytic, nuclear, photobiological, or photohybrid water electrolysis are being explored and offer the potential for a totally carbon-neutral process. Moreover, the use of wind turbines to drive water electrolysis and generate H_2_ is being tested as a feasible technology to store energy during off-peak hours.

Many microalgae have a H_2_-centered metabolism in which H_2_ serves as a source of reductant, and protons act as a sink for intracellular reductant under different environmental conditions. Of major interest, though, is the fact that microalgae are able to directly link photosynthetic water oxidation to H_2_ production by hydrogenases, thus holding the promise of plentiful energy from essentially inexhaustible sources—water and sunlight.

## Microalgae H_2_ pathways

As many other chlorophytes, the green unicellular alga *Chlamydomonas reinhardtii* is capable of producing H_2_ following a period of anaerobic induction (Gaffron and Rubin 1942; Healey 1970). Its genome is sequenced (Merchant et al. 2007), and many genetic and genomic tools to manipulate this organism are available. Indeed, *Chlamydomonas* is often called the “green yeast,” due to the power of its genetic system, while also having all the advantages of a microbe: facile genetics, flexible metabolism, and growth under heterotrophic, autotrophic, and mixotrophic conditions. As such, it has been used as a model organism to improve our understanding of H_2_ metabolism in microalgae and to provide a test bed for different hypotheses to optimize H_2_ production for commercial applications.

The photoproduction of H_2_ by *Chlamydomona*s is linked to photosynthesis, whereby light energy is converted into chemical energy as per the *Z* *scheme* (Ghirardi et al. 2009). In short, light absorbed by photosystem II (PSII) induces a charge-separated state involving P680^+^ and Pheophytin^−^ that extracts electrons from water, releasing O_2_ and protons into the chloroplast lumen. Concomitantly, light absorbed by photosystem I generates a strong oxidant P700^+^ that oxidizes an intermediate electron carrier (usually plastocyanin—PCY); the electron released from P700 reduces the electron acceptor ferredoxin (FDX). In linear electron flow (LEF), the electrons originated from PSII are transferred initially to plastoquinone (PQ) and, through a chain of carriers, reduce PCY. The final PSI electron acceptor, FDX, transfers electrons to the ferredoxin-NADP oxidoreductase (FNR) that in turn reduces NADP^+^ to NADPH, which is then consumed in the CO_2_ fixation reactions. Under anoxic conditions, FDX is also able to reduce the hydrogenases, catalyzing the reversible reduction of protons into molecular hydrogen (Florin et al. 2001).

There are three known hydrogen production pathways that contribute to H_2_ metabolism in *Chlamydomonas*. Two of those are mediated by the photosynthetic electron transfer chain, one being PSII dependent (direct pathway, described above) and the other PSII independent (indirect pathway). In the latter, reductant released from the glycolytic degradation of glucose are transferred through the enzyme NADP/plastoquinone oxidoreductase (NPQR) directly to the plastoquinone pool, bypassing PSII. On subsequent illumination, electrons are transferred down to the photosynthetic chain, reduce PCY, and are then reenergized by PSI and connected with the hydrogenase as in the direct pathway. Finally, the third H_2_-production pathway, which is linked to fermentation, is activated under dark anoxia and requires electron transfer from pyruvate to the hydrogenase through the pyruvate-ferredoxin-oxidoreductase (PFR). It is important to note that *Chlamydomonas* possesses two hydrogenases, HYDA1 and HYDA2 that can evolve H_2_ under anoxia through all of the three pathways (Meuser et al. 2012).

**Fig. 1:**
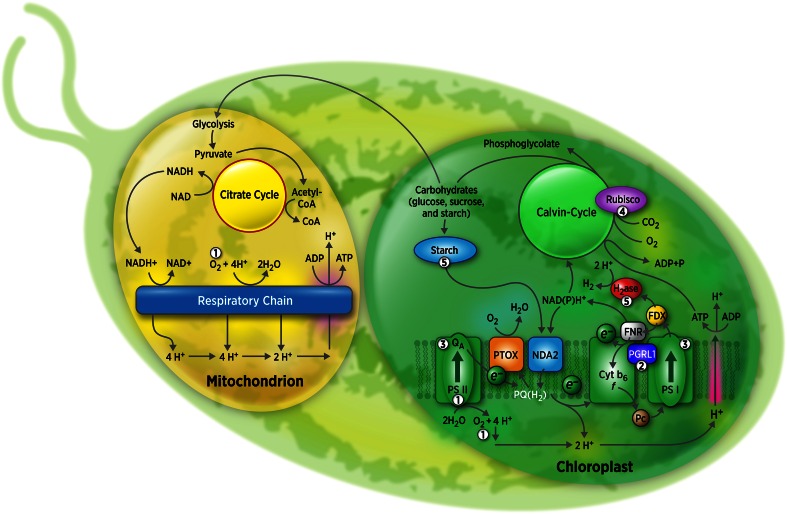
Representation of the hydrogen photoproduction-related pathways in *Chlamydomonas*. Hydrogen production occurs in the chloroplast, where the photosynthetic chain and the hydrogenases are located (see text for more details). The respiratory chain is located in the mitochondrion, and there is an extensive communication between the two organelles that can impact the level of hydrogen production (adapted from Kruse et al. 2005). The circled numbers indicate where current genetic engineering efforts have impacted H_2_ photoproduction, as described in the text. The barriers overcome by these modifications are: (1) O_2_ sensitivity, addressed by PSII inactivation and/or increased O_2_ consumption; (2) proton gradient dissipation, addressed by the *pgrl1* knockout mutation (decreased CEF); (3) photosynthetic efficiency, addressed by knockdown of light-harvesting antennae or truncating antenna proteins; (4) competition for electron, addressed by Rubisco mutagenesis; (5) low reductant flux and hydrogenase expression, addressed by impacting starch accumulation/degradation, FDX-HYD fusion, and overexpressing hydrogenase, respectively. It must be noted that, for clarity, not all the genetic engineering approaches mentioned in the text are represented in the figure

Although the potential energy conversion efficiency from sunlight to H_2_ by microalgae is theoretically high (about 10 %), H_2_ production is currently limited by biochemical and engineering constraints. Specific limitations include (a) the extreme sensitivity of the hydrogenases to O_2_; (b) low reductant availability for hydrogenase activity due to the existence of competing metabolic pathways that converge at the level of ferredoxin (FDX); (c) downregulation of photosynthetic electron transport and establishment of cyclic electron transfer around PSI under anaerobic, H_2_-producing conditions; (d) the low level at which light saturation occurs in photosynthesis; (e) the reversible nature of hydrogenases that results in consumption of H_2_ under high H_2_ partial pressure; and (f) the low levels of hydrogenase expression. Here we discuss in more detail each of the barriers mentioned above and describe the different genetic modification approaches that are being pursued to circumvent them and have led to improved hydrogen production (Fig. 1; Table 1).

**Table 1:**
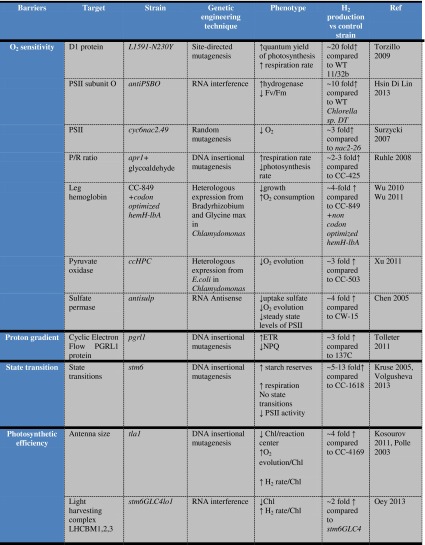

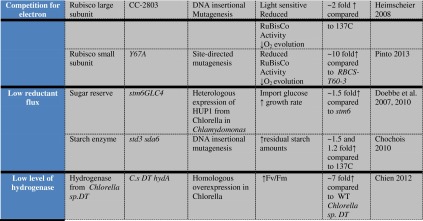
Summary of the genetically engineered strains with improved H_2_ production

For more details, refer to the text and references (adapted from Esquível et al. 2011). *Note* We followed the nomenclature set by the www.chlamy.org website for eukaryotic genes throughout the text. Genes are listed: uppercase letters, italics (nuclear encoded) or lowercase with the last letter uppercase, italics (chloroplast encoded); proteins in uppercase letter, no italics; mutant strains in lowercase, italics. Prokaryotic nomenclature is set as follow: Genes and mutant strains are listed in lowercase with the last letter uppercase, italics; proteins: first and last letter capital, italics

## Barriers

### O_2_ sensitivity of hydrogenases

Anaerobiosis is a prerequisite for H_2_ production by algae. Indeed, *Chlamydomonas* cultures are capable of photoproducing hydrogen at a very high efficiency (close to the maximal photosynthesis yield ~10 %) for a few minutes upon illumination. The process stops due to, among other barriers, the inactivation of the hydrogenase by O_2_ generated at PSII. Various approaches have been utilized to overcome this inactivation (see “Genetic engineering to overcome limitations to hydrogen production” section below). The most successful one is based on the selective inactivation of PSII O_2_ evolution activity by sulfur deprivation (Melis et al. 2000). The sulfur-deprived system is usually operated in two stages. In the first stage, sulfur-deprived and illuminated cultures gradually inactivate PSII (the absence of sulfur prevents repair of photodamaged PSII) and simultaneously overaccumulate starch. When the rate of O_2_ photoproduced by PSII matches the rate of O_2_ consumption by respiration, the cultures become anaerobic. During the second stage, the residual PSII activity and concomitant starch degradation supply reductant to the photosynthetic chain through the operation of the direct and indirect electron transport pathways (Posewitz et al. 2005) and enable H_2_ photoproduction to occur. This approach, although convenient for laboratory studies, is, however, not scalable for commercial purposes due to its low inherent conversion efficiency (James et al. 2008).

Other approaches to circumventing the O_2_-sensitivity problem require either engineering an O_2_-tolerant algal [FeFe]-hydrogenase (Chang et al. 2007) or expressing a hydrogenase that is more tolerant to O_2_ in *Chlamydomonas*. Molecular dynamics simulations, solvent accessibility maps, and potential mean energy estimates have been used to identify gas diffusion pathways in model enzymes (Chang et al. 2007), followed by site-directed mutagenesis (Long et al. 2009). However, this approach has not been successful due to the unexpected observation that the amino acid residues responsible for binding of the catalytic cluster are also involved in the formation of the gas channels (Mulder et al. 2010). Thus, mutants affecting these residues are unable to properly fold the protein. This observation explains the lower activity and higher O_2_ sensitivity of mutants that were generated based on the information provided by the computational models (Liebgott et al. 2010).

### Non-dissipated proton gradient and state transitions

The anaerobic treatment used to induce H_2_ production in both sulfur-replete and -depleted cultures triggers starch degradation, causing reduction of the PQ pool through the NPQR enzyme. These conditions poise the cultures in state 2 and, upon illumination, trigger the CEF mode—which contributes to an increase in the proton gradient that normally drives ATP synthesis through the ATP synthase enzyme. In state 2, a fraction of the light-harvesting antenna of PSII gets connected to PSI, increasing its light-absorption cross section at the expenses of that of PSII and supposedly increasing CEF over LEF. However, since H_2_ photoproduction does not consume ATP, the proton gradient will remain undissipated when the anaerobically induced cells are illuminated. The non-dissipated proton gradient induces non-photochemical quenching mechanisms that lower the efficiency of photosynthetic electron utilization and thus of H_2_ production. The evidence for the effect of the non-dissipated proton gradient in H_2_ production is supported by the observation that proton uncouplers stimulate the rates of H_2_ photoproduction in sulfur-replete (Happe et al. 1994) and sulfur-depleted conditions [(Tolleter et al. 2011)—see “Barrier: proton gradient” section for further discussion]. Moreover, the influence of state 2 on downregulation of H_2_ production was confirmed by the recent report of a mutant locked in state 1I, *stm6* (discussed in “Genetic engineering to overcome limitations to hydrogen production” section) that showed higher rates of H_2_ photoproduction than its parental strain (Kruse et al. 2005).

### Small antenna size

As true of other photosynthetic processes, the efficiency of photohydrogen production by mass cultures under solar intensity is limited by the large antenna size of the photosystems. Under high light fluxes, the photons absorbed by the light-harvesting antennae of PSI and PSII are underutilized and are dissipated as fluorescence or heat. Thus, in a high-density mass culture, cells at the surface overabsorb and waste sunlight; whereas cells deeper in the culture are deprived of light due to shading. The photosynthetic capacity of the cell is, therefore, not used at its maximum potential.

### Competition for photosynthetic reductant

Algal H_2_ production is also limited by the existence of pathways that compete directly with the hydrogenase for photosynthetic reductant from ferredoxin. These include FNR, FTR (ferredoxin/thioredoxin reductase), nitrite reductase, sulfite reductase, and glutamate synthase. The activities of all these enzymes do have an impact on hydrogen production, since they decrease the electron flux toward hydrogenase depending on the physiological conditions in the cell. In *Chlamydomonas*, only two out of the six chloroplast-localized ferredoxins (FDXs), FDX1 and FDX2, are functionally linked to the hydrogenases. These two FDXs share similar binding partners but FDX1 serves as the primary electron donor to three important biological pathways, NADP^+^ reduction, and H_2_-photo and fermentative production. FDX2 is also capable of driving these reactions but at less than half the rate observed for FDX1 (Noth et al. 2013; van Lis et al. 2013; Peden et al. 2013). Finally, FDX1 is also involved in transferring electron to PGRL1, the protein that mediates cyclic electron transfer through the Cyt b6/f complex.

## Genetic engineering to overcome limitations to hydrogen production

Recent genetic engineering efforts have pushed forward the biohydrogen research area and provided additional insight into the complex interaction among the diverse pathways involved in the process. Next, we discuss some of the genetically modified strains that led to improved hydrogen production (see Table 1 for a summary of strain phenotypes).

### Barrier: O_2_ sensitivity

Many attempts have been made to generate O_2_-tolerant hydrogenases through random mutagenesis in vivo (Ghirardi et al. 1997) and in vitro (Stapleton and Swartz 2010). Unfortunately, these efforts yielded only small changes in O_2_ tolerance. As an alternative approach, various research groups developed different methods to induce anaerobic conditions, either by partially inactivating PSII in order to decrease the rates of O_2_ evolution (as achieved by sulfur deprivation) or to increase O_2_ uptake/sequestration within the cell.

#### Partial PSII inactivation

The D1 protein is part of the PSII reaction center and, together with D2, binds the majority of the cofactors involved in the PSII-dependent electron transport. Most of the amino acid residues between S155 and D170 in D1 (Ohad and Hirschberg 1992; Lardans et al. 1998; Xiong et al. 1998) appear to be crucial in mediating electron transfer from the D1-Y161 (or donor Z) to P680^+^ (Hutchison et al. 1996), and some of them (e.g., D170) have been demonstrated to be crucial for binding the manganese cluster (Ohad and Hirschberg 1992; Nixon and Diner 1992; Chu et al. 1995). They are thus promising targets for mutagenesis aimed at inactivating PSII activity. The phenotypic characterization of the L1591-N230Y mutant in *Chlamydomonas* was recently reported (Scoma et al. 2012; Torzillo et al. 2009). This mutant has lower chlorophyll content, higher photosynthetic capacity, and higher relative quantum yield of photosynthesis, together with higher respiration rate and a very high conversion of violaxanthin to zeaxanthin during H_2_ production, suggesting better photoprotection under high light. This strain produced 20 times more H_2_ than the wild-type strain and for longer periods of time, thus validating the concept that partial PSII inactivation promotes higher H_2_-production activity.

Partial inactivation of O_2_ evolution was also reported in *Chlorella* sp. DT, and it was achieved by knocking down the *PSBO* subunit of PSII. The authors used short interference RNA antisense-*PSBO* fragments and observed that the *HYDA* gene transcription and the HYDA expression levels were increased in the *psbo*-knockdown mutants (Lin et al. 2013). Under low illumination and semi-aerobic conditions (the *Chlorella* native hydrogenase has increased tolerance to O_2_), they reported that photobiological H_2_ production increased by as much as tenfold compared to its WT (Lin et al. 2013).

Recently, a genetic switch was developed to regulate PSII activity and allow control of the oxygen level and electron flux in the cell (Surzycki et al. 2007). The switch is composed of the nuclear-encoded NAC2 chloroplast protein that is required for the stable accumulation of the *psbD* RNA (which encodes the PSII D2 reaction center protein), and the anoxia-dependent copper-sensitive cytochrome *CYC6* promoter. A construct containing the two fused DNA sequences was used to control the expression of the D2 protein in transgenic strains. Under aerobic conditions, expression of *NAC* *2* is repressed, and the D2 protein becomes unstable, causing gradual loss of O_2_-evolution activity and leading to anaerobiosis and H_2_ production by the organism. The resulting anaerobic conditions induce re-synthesis of NAC2 protein and result in increased stability of D2; under these conditions, O_2_ evolution is gradually restored and H_2_ production is inhibited. The alternate expression and repression of the *NAC2* gene, without the need for removing copper from the medium serves the same purpose as the sulfur-deprivation process described in “O_2_ sensitivity of hydrogenases” section, allowing the operation of a 2-phase system where carbon reserves accumulate during the oxygenic phases and subsequently support the respiratory activity needed to achieve anaerobiosis during the second phase. The authors report the production of 20 μmol H_2_/liter during one cycle, which corresponds to a maximal rate of 1 mmol H_2_ mol^−1^ Chl s^−1^.

#### Increased O_2_ consumption/sequestration

Anaerobiosis can be achieved either by decreasing O_2_ evolution or increasing respiration, i.e., by manipulating the photosynthesis/respiration ratio (P/R ratio) and bringing it below 1. The *apr1* mutant, which exhibited an attenuated P/R ratio (Ruhle et al. 2008), was shown to become anaerobic in the light, mimicking the physiological status of sulfur-deprived cells. In this strain, starch is degraded under non-stress conditions and the reducing equivalents are transferred by the NAD(P)H plastoquinone-oxidoreductase (NPQR, also called NDA2) to the plastoquinone pool (PQ) (Mus et al. 2005), keeping it reduced. As a consequence, CEF and photophosphorylation still occur, although PSII activity is substantially downregulated. The *apr1* mutant becomes anaerobic under photoheterotrophic, sulfur-replete conditions and induces hydrogenase synthesis in the light. However, it does not produce hydrogen, contrary to expectations. In the past, it has been shown that hydrogen production in anaerobically adapted algae is highest when the carbon dioxide concentrations are low (Cinco et al. 1993), due to competition between hydrogenase and FNR for photosynthetic reductant. Photoreduced FDX transfers electrons mainly to FNR, which then supplies NADPH to the Calvin–Benson Cycle. Thus, to disrupt the effect of the Calvin Benson cycle activity on hydrogen metabolism, glycolaldehyde (GA) was added to the *apr1* culture. GA disrupts the Calvin–Benson cycle activity by inhibiting the phosphoribulokinase, which catalyzes the ATP-dependent phosphorylation of ribulose-5-phosphate to ribulose-1,5-bisphosphate. Consequently, it was observed that the in vivo hydrogen production rate of *apr1* cell samples was twice the rate determined in WT sulfur-deprived cells, thus confirming the usefulness of the low P/R ratio concept (Ruhle et al. 2008).

Another approach to induce anaerobiosis is by introducing O_2_ sequesters into the chloroplast. Leghemoglobins (LbA) proteins sequester and carry O_2_ from the legumes’ root nodules to symbiotic N_2_-fixing Rhizobia in order to keep the O_2_ levels low around their nitrogenase. The maturation of leghemoglobins requires the rhizobial *hemH* gene that encodes for a ferrochelatase, that is necessary for catalyzing the last step of heme synthesis (Frustaci and O’Brian 1992). Wu et al. 2010 cloned the *hemH* and the *lbA* genes as a fusion construct, transformed them into the chloroplast of *Chlamydomonas*, and demonstrated that the expression of the respective fusion protein improved H_2_ yields by decreasing the O_2_ content in the medium; both in the presence and absence of sulfur H_2_ yields in transgenic algal cultures increased, to as much as fourfold in sulfur-free medium compared to the wild type, correlating to the highest expression levels of the HemH-LbA fusion protein in the cell. To further improve their yield, the authors generated a codon-optimized construct of the *hemH* gene and observed that the expression level of HemH-LbA protein increased 6.8-fold in the transgenic alga compared with the non-codon-optimized strain, resulting in a 22 % increase in the H_2_ yield and an overall increase of 134 % in O_2_ uptake compared to the control WT cultures (Wu et al. 2011).

Alternative approaches to remove O_2_ from the culture medium include the introduction of new pathways in *Chlamydomonas* that utilize O_2_. The enzyme pyruvate oxidase (PoX) catalyzes the decarboxylation of pyruvate to acetyl phosphate and CO_2_. Since this reaction requires O_2_, it was hypothesized that introducing this gene in *Chlamydomonas* could help decrease the intracellular O_2_ levels (Xu et al. 2011). In *E. coli*, pyruvate oxidase plays an important role in aerobic growth by maintaining the pool of free CoA (Flores et al. 2004). The transgenic alga expressing the *E. coli* *poX* showed low oxygen evolution and no defect on growth rate. Moreover, it was capable of producing hydrogen at twice the rate of its WT (Xu et al. 2011).

Finally, to recreate the effect of sulfur depletion in the cell, an antisense technology was applied to *Chlamydomonas* to probe the effect of the repression of the sulfate permease gene, *SULP*. As expected, the anti*sulp* transformants were impaired in sulfate uptake, and exhibited a sulfur-deprivation phenotype, with strong induction of arylsulfatase activity and global induction of the expression of sulfate assimilation genes. The cells displayed slower rates of light-saturated oxygen evolution, lower levels of Rubisco, and lower steady-state levels of the PSII D1 reaction center protein, suggesting that attenuation of the SulP gene expression immediately affects the repair of PSII from photo-oxidative damage (Chen et al. 2005). The expression of the *SULP* gene also led to a lowering in PSII activity, establishing anaerobiosis more quickly in the cell. Under anaerobiosis, the anti*sulp* strains produce less oxygen and photoevolve H_2_ (Chen et al. 2005).

In our view, methods based on partial inactivation of PSII by itself will not achieve high light-conversion efficiencies (James et al. 2008), even if they are combined with increased respiratory O_2_ consumption. However, the effect of expressing O_2_ sequesters, such as leghemoglobin and the pyruvate oxidase enzyme, in *Chlamydomonas* should be analyzed more carefully to determine (a) the total O_2_-binding capability of leghemoglobin molecules, and how the O_2_ is eventually released to the medium, and (b) the efficacy of the pyruvate oxidase reaction in long-term, high-H_2_-producing conditions. An additional approach under consideration involves the expression of one of the clostridial [FeFe]-hydrogenases in *Chlamydomonas*. These enzymes have been shown to have two orders of magnitude higher tolerance to O_2_ in vitro, and one needs to verify whether it maintains its higher O_2_ tolerance when physiologically connected to the *Chlamydomonas* photosynthetic apparatus as well.

### Barrier: proton gradient

The downregulation of photosynthetic LEF by non-dissipation of the proton gradient in H_2_-producing cell was addressed by isolation of a mutant deficient in PGRL1, as described in “Non-dissipated proton gradient and state transitions” sections. The PGRL1 protein is a component of a supercomplex that includes PSI-LHCI-LHCII-FNR-Cytochrome b6/f; this supercomplex is proposed to mediate CEF, and its operation is induced by high light conditions. When PGRL1 is genetically disrupted, the CEF around PSI becomes non-operational (Tolleter et al. 2011). The *pgrl1* mutant strain was shown to exhibit lower CEF and increased hydrogen production under both short-term (argon-induced) and long-term (sulfur-deprivation-induced) anaerobiosis under high light. The authors concluded that the proton gradient generated by CEF in WT cells under high illumination strongly limits the electron supply to hydrogenase, and it can be overcome by disrupting components of the supercomplex. Moreover, as expected, the mutant strain exhibited reduced NPQ, likely resulting from the decrease in the CEF-dependent proton gradient.

Although it has been shown recently that state transitions do not control CET (Lucker and Kramer 2013; Takahashi et al. 2013), a mutant blocked in state 1 (*stm6*) showed no CET, higher respiratory metabolism, large starch reserves, and a low dissolved O_2_ concentration (40 % of the wild type (WT)), resulting in increased hydrogen production following anaerobic induction. No direct effect on PSII activity was reported, possibly due to the fact that anaerobiosis could be achieved faster—thus protecting PSII from irreversible photoinhibition. The H_2_-production rates of were 5–13 times higher than the control WT strain over a range of conditions (light intensity, culture time, and addition of uncouplers). More recent studies demonstrated that most PSII centers are “closed” in the *stm6* mutant during the anaerobic phase, and that, under sulfur-deprivation conditions, water splitting by the remaining open PSII supplies the majority of electrons for H_2_ synthesis (Volgusheva et al. 2013).

Both of the mutants described above are currently being genetically combined with strains expressing other traits that overcome additional barriers, such as truncated antennae (see “Barrier: photosynthetic efficiency” section). However, we must point out that the host strain used to generate the *stm6* mutant is a low H_2_ producer compared to other *Chlamydomonas* WT strains such as  CC-124 and D66. It would be more useful if the *stm6* mutant genotype were genetically transferred to one of these high H_2_-producing WT strains to increase the chance that it will achieve higher conversion efficiencies in the future.

### Barrier: photosynthetic efficiency

The concept of decreasing the chlorophyll antenna size of the photosystems to increase the light utilization efficiency of algal mass cultures has been proposed in the past (Melis et al. 2000; Melis and Chen 2005). Research efforts to test it have focused on using random mutagenesis and high-throughput screening to aid the identification of genes that regulate the Chl antenna size in green alga. This work has resulted in strains with gradually smaller antenna sizes and increasing photosynthetic productivity (Polle et al. 2003; Tetali et al. 2007; Mitra and Melis 2010; Kirst et al. 2012a, b). Analysis of the *Chlamydomonas* *tla1* truncated antenna mutant proved that the concept is also successful in increasing H_2_ productivity. Kosourov et al. 2011 immobilized WT and *tla1* sulfur-deprived mutant cells on alginate fims and monitored long-term H_2_-photoproduction activity under light intensities ranging from 19 to 350 μE m^−2^ s^−1^PAR. They showed that the mutant was able to produce H_2_ gas for over 250 h under all light conditions tested and exhibited a 4–8 times higher maximum specific rate between 285 and 350 μE m^−2^ s^−1^, compared to WT cells.

Along the same line, RNAi knockdowns of the light-harvesting complexes M1, 2, and 3 were performed to reduce the antenna size and optimize light capture by *Chlamydomonas*. LHCBM1, 2, and 3 are known to be the most abundant LHC proteins, and knocking them down simultaneously reduced the total chlorophyll content of the cells—resulting in improved light penetration and utilization. This multiple mutant displayed higher photosynthesis light saturation level and did not suffer photoinhibition under saturating light intensity. Upon sulfur deprivation, the mutant strain showed an immediate onset of H_2_ production, indicating that the intracellular O_2_ levels were already poised to induce *HYDA* transcription. Furthermore, the rate of H_2_ production observed in this strain was twice as high as that of the *stm6GLC4* (Oey et al. 2013) described below.

As mentioned in the previous section, both the *tla* and the *lhcb* mutants are being or have been introduced into strains that are not limited by the non-dissipation of the proton gtradient and will continue to serve as the host for other strains expressing additional useful traits.

### Barrier: competition for electrons

The major pathway competing for photosynthetic reducing power with the hydrogenase is CO_2_ fixation, whose first step is NADP^+^ photoreduction by FNR. Under oxic conditions, most of the photosynthetic reductant is directed from FDX1 to FNR—which produces NADPH. When the cells become anoxic, HYDA competes with FNR at the level of FDX1. In order to reduce this competition (and bypass the dominating effect of FNR), a ferredoxin-hydrogenase fusion was engineered and tested in vitro (Yacoby et al. 2011). It was shown that the H_2_-photoproduction activity of the fusion was sixfold higher than that using isolated HYDA and added FDX. The authors proposed that the fusion successfully insulates FDX1 internal electrons from exogenous competitors, and demonstrated that only 10 % of the photosynthetic electrons are lost to FNR in the absence of added FDX. Finally, they showed that the fusion was able to overcome NADP^+^ competitive inhibition, as more than 60 % of photosynthetic electrons were diverted to hydrogen production compared to less than 10 % for non-fused HYDA (Yacoby et al. 2011).

The subsequent steps in CO_2_ fixation involve the carboxylation of ribulose *bis*-phosphate by the enzyme Rubisco. This enzyme plays an important role in the global carbon cycle and photorespiratory oxygen consumption. Thus, not surprisingly, strain CC-2803, which is impaired in CO_2_ fixation (lacking the large subunit of Rubisco), showed a higher rate of H_2_ production than its wild-type parent under sulfur deprivation (Hemschemeier et al. 2008). Similarly, an engineered *Chlamydomonas* strain harboring a mutation on tyrosine 67 of the Rubisco small subunit displayed 10- to 15-fold higher hydrogen production rate than its WT (Pinto et al. 2013). This latter mutation was shown to impair the stability of Rubisco (Esquivel et al. 2006) and resulted in a decrease in efficiency and the amount of PSII protein complexes (Pinto et al. 2013). The phenotype was explained by the feedback inhibitory effect of eliminating a major electron sink on the generation of reductant/protons by PSII (Skillman 2008). It is also known that inhibition of the Calvin Cycle leads to over-reduction of the photosynthetic electron transport chain, thus promoting the generation of reactive oxygen species in PSII, which may have caused increased photoinhibition (Antal et al. 2010).

### Barrier: low reductant flux to the hydrogenase

As mentioned above, in the presence of active CO_2_ fixation, the reductant flux available for hydrogen production is low. In order to increase this flux, a HUP1 (hexose uptake protein) hexose symporter from *Chlorella kessleri* was incorporated into the *Chlamydomonas* *stm6* mutant strain (Doebbe et al. 2007). The rationale was to develop a strain capable of providing additional reductant to the hydrogenase by increasing the amount of respiratory substrate. This new engineered strain can use externally supplied glucose for heterotrophic growth in the dark. In the light, a 1.5-fold increase in H_2_-production capacity was observed. Coupling an external carbon source to H_2_ synthesis, thus, represents an alternative for feedstock utilization and fuel production (Doebbe et al. 2007).

Starch metabolism is an important factor for hydrogen production, since it is the source for reductant to the PSII-independent (or indirect) pathway. To better understand the impact of starch degradation on hydrogen production, a mutant library was developed and screened for mutants affected in starch catabolism (Chochois et al. 2010). The results showed that mutants with the strongest impact on starch catabolism generally displayed lower hydrogen production by the PSII-independent pathway than their parental strains. On the other hand, while mutants that were only slightly affected in starch degradation exhibited a delay in their H_2_-production activity under sulfur deprivation. Two mutant strains showed a much higher total hydrogen production yield than the wild type, although they displayed different phenotypes. In the first, *std* *3*, the amount of starch accumulated under sulfur deprivation was similar to the wild type but the % of residual starch left at the end of the H_2_-production phase was lower—suggesting that faster degradation kinetics correlated with higher hydrogen production. The second mutant, *sda* *6*, showed a slow rate of starch degradation, accompanied by an initial H_2_-production rate that was lower than the WT; however, the final H_2_ yield was much higher than that of the WT. These studies support the relationship between the indirect hydrogen production pathway and starch catabolism, and emphasize the importance of its contribution to overall algal H_2_ photoproduction—signaling an alternative method to manipulate algal H_2_ production (Chochois et al. 2010).

Although experimental evidence demonstrates that overall H_2_-production rates increase in the presence of exogenous or higher endogenous levels of organic substrate, it is not clear whether this approach would result in a more cost-effective process, given that either (a) the cost of the organic substrate will increase the overall cost of the process or (b) the organism will have to undergo the sulfur-deprivation process to induce endogenous carbon substrate catabolism and hydrogenase activity—which has been shown to have overall unsatisfactory light-conversion efficiency (James et al. 2008).

It must be noted that the low level of hydrogense gene expression or the rapid turnover of the protein due to presence of oxygen was also proposed to contribute to the low level of H_2_ production. Homologous overexpression of the *Chlorella* sp. DT hydrogenase shows that it is possible to increase hydrogen production by overexpressing the enzyme. This alga contains a hydrogenase that is more oxygen tolerant than the *Chlamydomonas* enzyme, and is capable of producing small amounts of hydrogen under aerobic and sulfur-replete conditions. The overexpression of this enzyme in the native host led to 7- to 10-fold increase in hydrogen production yield (Chien et al. 2012).

This is a promising approach that could be attempted in high H_2_-producing algae, such as *Chlamydomonas* or combined with methods to sequester photosynthetically evolved O_2_ in *Chlorella*.

## Additional potential bottlenecks in hydrogen production

Biological hydrogen photoproduction is a complex process that requires a tight control/regulation of many pathways at different levels. Genetic engineering has been employed to overcome these limitations and, in most cases, hydrogen production rates have been improved. However, additional genetic modification will be required to achieve maximal conversion efficiency of solar energy into biohydrogen. These include but are not limited to (a) designing an inducible leaky ATP synthase mutant and/or inducible proton channel, whereby the proton gradient is dissipated while the cell produces H_2_; (b) increasing the size of the PQ pool to ameliorate the rate-limiting step in photosynthetic electron transport, the oxidation of the PQ pool; and (c) overexpressing NDA2 to increase electron flux into and from the indirect hydrogen production pathway.

## High-throughput screening techniques

To screen for mutants altered in H_2_ production, several techniques have been developed in the past years as described below. One of the best available methods is a solid-state chemochromic H_2_ sensor consisting of tungsten oxide and palladium. The palladium captures H_2_ and transfers it to the tungsten oxide which turns blue when reduced. *Chlamydomonas* insertional mutants plated on Petri dishes were screened for attenuated hydrogen production following induction in an anaerobic glove box overnight. When exposed to the light, the cells photoevolved H_2_, which was detected as blue dots on the H_2_ sensor (Seibert et al. 2001; Flynn et al. 2002). This method was successfully used to identify the hydrogenase catalytic cluster assembly genes *HYDEF* and *HYDG* (Posewitz et al. 2004a) and a starch-less mutant, *sta7*, in which hydrogenase gene transcription is repressed (Posewitz et al. 2004b).

A water-soluble color indicator has also been used to screen hydrogen-producing microorganisms. This indicator consists of a coloring agent and a water-soluble derivative of Wilkinson’s catalyst [Tris(triphenylphosphine) rhodium chloride]. In this screen, methyl orange and the sulfonate catalyst are dissolved in water and change color when in contact with hydrogen gas. This system can be used with any H_2_-producing microorganism (Katsuda et al. 2006).

**Fig. 2:**
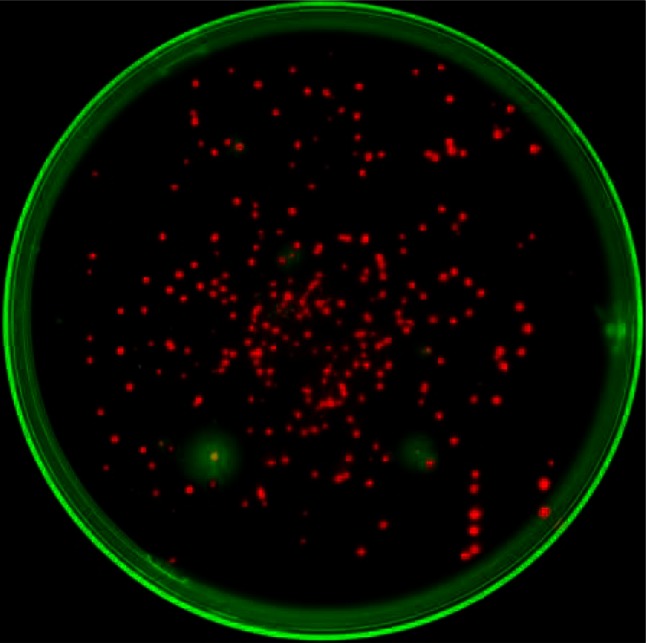
Detection of H_2_ photoproduction by algal colonies at high light fluxes using the *R. capsulatus* emGFP overlay screening assay. Composite images indicating H_2_ production in *green* and colony density in *red*, as taken with a Fluorchem Q imaging system, are shown. Transformants from a *Chlamydomonas reinhardtii* insertional mutagenesis library were plated on hygromycin plates, and overlaid with the *Rhodobacter capsulatus* GFP-based H_2_-sensing system. The plate was incubated for 16 h at 300 μE m^−2^ s^−1^ light prior to fluorescence imaging. The figure shows four strains capable of H_2_ production at this light level (Wecker et al. 2011)

Finally, a new and very sensitive technique was recently developed, based on the sensing system from *Rhodobacter capsulatus*—which acts to upregulate the expression of the native cell’s uptake hydrogenase in response to H_2_. The *Rhodobacter* system is composed of the H_2_-sensor protein (HupUV), a histidine kinase (HupT), a transcription regulator (HupR), and an uptake hydrogenase (HupSL). In the absence of H_2_, the sensor HupUV interacts with the kinase HupT inducing its autophosphorylation (Elsen et al. 1993). The activated kinase then phosphorylates the HupR regulator which downregulates the expression of the uptake hydrogenase. In the presence of H_2_, the kinase and the regulator proteins remain dephosphorylated, and the HupR regulator binds to and activates the S70 RNA polymerase-(RNAP)-dependent transcription of hupSL. The regulator *hupR* is constitutively expressed at low levels in *R. capsulatus* (Dischert et al. 1999), whereas both *hupUV* and *hupT* are transcriptionally regulated from the *hupT* promoter and are transcribed at levels 50-fold lower than *hupR* (Vignais et al. 1997). Wecker et al. 2011 developed a screen in which the emGFP reporter protein is integrated behind the *hupSL* promoter of *R. capsulatus*. Hydrogen-sensing *R. capsulatus* cells were grown fermentatively in the dark in co-culture with *Chlamydomonas* on microtiter plates and the bacteria fluoresced in response to H_2_ production by the algae. The H_2_-producing algal cells are easily visualized for H_2_ induction, respond to as little as 200 pM H_2_ in solution (0.33 ppm by volume in the headspace), and do not need to be lysed. This in situ H_2_-production detection system has been adapted to light-induced high-throughput analyses, and was shown to discriminate among a diversity of H_2_-production phenotypes (Wecker and Ghirardi 2014; Fig. 2).

## Molecular and metabolic engineering: what tools are available?

Despite its use in algal research for several decades, *Chlamydomonas* remains a difficult platform for conducting genetic alterations. Genetic engineering relies on the expression of transgenes inserted at random into the genome via illegitimate recombination. The lack of tools for targeted gene insertion in green algae is a major impediment to the rapid progress of biological hydrogen production. Nuclear gene targeting and site-directed mutagenesis will be necessary to achieve fine-control over the hydrogen production machinery. A more controlled system would require replacement of the target gene via homologous recombination, which would enable *Chlamydomonas* to become a technical platform for the research community. Novel approaches are being developed to facilitate gene targeting, such as Cas9-based CRiSPR and knockouts of non-homologous pathways, as previously done in yeast (DiCarlo et al. 2013).

## Integrated strategy

It is clear that some of the most advantageous traits described in “Genetic engineering to overcome limitations to hydrogen production” and “Additional potential bottlenecks in hydrogen production” sections need to be incorporated into a single green algal strain to evaluate their additive effect on the rates and yields of H_2_ photoproduction. The use of genetics to cross different mutant lines should play an increasing role in further development of this technology. In our view, a mutant expressing a more O_2_-tolerant hydrogenase, such as the *Clostridium acetobutylicum* Ca1, the *pgrl1* mutation, a truncated antenna, and an inducible Fd/hydrogenase fusion, represents one of the most promising genetic combinations to achieve long-term high-efficiency H_2_-producing activity, at this juncture. Obviously, other mutant constructs, containing for instance O_2_ sequesters and other proton gradient dissipators, are equally promising and worth pursuing. This research area is expanding rapidly, based on the premise and promise of a cost-effective carbon-neutral energy technology.
